# Health knowledge after stroke in Switzerland: a survey among health professionals on current practice and suggestions for the implementation of a technology-based educational program for stroke survivors

**DOI:** 10.1186/s12913-024-11735-0

**Published:** 2024-10-19

**Authors:** Giada Devittori, Mosè Peduzzi, Daria Dinacci, Paolo Rossi, Claudio Petrillo, Roger Gassert, Olivier Lambercy

**Affiliations:** 1 Rehabilitation Engineering Laboratory, Swiss Federal Institute of Technology Zürich, Zurich, Switzerland; 2 Clinica Hildebrand Centro di Riabilitazione Brissago, Brissago, Switzerland; 3 Future Health Technologies, Singapore-ETH Centre, Campus for Research Excellence and Technological Enterprise (CREATE), Singapore, Singapore

**Keywords:** Health literacy, Health education, Stroke education, Stroke, Secondary prevention, Educational program, Technology-based education, Technology-assisted rehabilitation

## Abstract

**Background:**

It is estimated that 1 in 4 stroke survivors will experience a second stroke. Educating patients about risk factors for stroke and a generally healthier lifestyle may help prevent recurrent strokes, which are a burden on society and the healthcare system. The goals of this paper were to investigate the estimated level of knowledge of stroke patients regarding their disease, the methods of information commonly used in clinical practice, the topics that should be included in an educational program aimed at improving health knowledge among stroke survivors, and how such a program could be delivered with the help of technology-based education (i.e., information delivered by digital platforms such as smartphones or rehabilitation technologies).

**Methods:**

We performed a survey among health professionals working with stroke patients in Switzerland.

**Results:**

161 health professionals of different backgrounds took part in the survey, and 94 completed it. According to the results, only 33% of healthcare professionals thought that patients were well informed about stroke one month after stroke onset. These findings suggest that there is room for improvement in how stroke patients are educated about stroke, risk factors, and prevention. Additionally, it was highlighted that technology is not commonly used in clinical practice to support patients’ education, although this is an acceptable method for healthcare professionals. The results also helped to identify key topics to be included in an educational program and recommendations for implementing such a program in rehabilitation technologies.

**Conclusions:**

This work allowed gaining more insight into healthcare professionals’ opinions on the potential of technology-based education and key aspects to consider when implementing it to support health and prevention knowledge after stroke.

**Supplementary Information:**

The online version contains supplementary material available at 10.1186/s12913-024-11735-0.

## Background

In Switzerland, approximately 20’000 people suffer a stroke each year [1], and this number has been increasing [2, 3]. Moreover, the risk of a recurrent stroke five years after the first event is roughly 25% [4]. Addressing recurrent strokes is thus critical, as they raise costs for the healthcare system [5] and further decrease patients’ quality of life. An important means of decreasing the number of recurrent strokes is secondary prevention [6, 7], which includes the implementation of different strategies to reduce risk factors for stroke [8]. Educating individuals after a stroke about modifiable and behavioural risk factors (e.g., unhealthy diet, smoking) is, therefore, indispensable to minimize them [6, 9]. Additionally, higher health literacy is generally associated with better rehabilitation outcomes and quality of life post-stroke [10]. Furthermore, providing medical information about stroke to stroke patients might also lead to higher adherence to therapies [11]. However, stroke patients’ knowledge about risk factors, health, treatment options, and other relevant topics is thought to be suboptimal according to studies performed in Switzerland [12] and other parts of the world [13, 14].

To address this shortcoming, there exist different methods aimed at improving health literacy among stroke survivors. Some methods are based on the interaction with healthcare professionals, as for instance the oral transmission of information during one-to-one or group sessions [15–17]. The advantages of these methods are that healthcare professionals are usually experienced in delivering information and can adapt according to the reactions of the patient, meaning that the provided information can be individualized, while also providing patients with the possibility to ask questions. However, such sessions often take place in hospitals or clinics, which might not be ideal for out-patients, their frequency can be limited by the time availability of the healthcare professionals, they are not standardized across different institutions, and the information provided can be perceived as too difficult to understand due to the use of medical jargon [18]. Self-education with the support of brochures, books, journals, or the internet might be another option [19, 20], the advantage here being that these tools can be accessed anytime and from anywhere and do not require additional time from the healthcare professionals. Such approaches may help reduce the problem related to non-retention of information when given only once or too soon immediately after the stroke [20]. Nevertheless, the information accessed via these tools is not personalized and the content might be too difficult to understand, misleading, or not trustworthy.

In recent years, digital technologies (e.g., mobile applications [21–23], videos [24], systems for telerehabilitation or unsupervised therapy [25, 26]) have been proposed as a promising tool to support health education [27, 28] and overcome the existing limitations. Technology-based unsupervised education, meaning the delivery of information via educational programs implemented into technologies used by patients independently, might be a valid alternative to the methods described before. This is because it has the potential to scale up the delivery of personalized and controlled information without increasing the workload for healthcare professionals while remaining accessible to the patients. Furthermore, there are additional advantages when the educational program is implemented in combination with a rehabilitation technology (e.g., robotic devices, telerehabilitation systems). For example, a systematic administration of the desired information is possible through the implementation of reminders or the active presentation of the information during a rehabilitation session [25, 26]. However, there are no widely accepted guidelines on how to implement such an educational program (e.g., type and format of the information) and, due to the emerging nature of technology-based health education, it is not clear what the opinion of healthcare professionals is about this approach.

Therefore, in order to better understand how the health education of stroke patients is handled and how technologies could help, we conducted a survey among healthcare professionals in Switzerland. We chose to focus on a single country due to country-specific differences in healthcare systems and educational resources available, which would make them difficult to compare. The aims of the survey were the following: (i) to gather the opinion of healthcare professionals working in Switzerland about the level of education of stroke patients regarding their disease, (ii) to investigate how stroke patients are informed in clinical practice (i.e., methods used), (iii) to identify topics that should be covered in an educational program, as well as to (iv) evaluate if education assisted by rehabilitation technologies would be feasible and explore possible pathways for its implementation. This work is important as it will help to define key factors that need to be considered when developing novel tools to improve the communication of health-related information after stroke, according to healthcare professionals.

## Methods

This study was approved by the ethics committee of the Swiss Federal Institute of Technology (ETH) of Zurich (EK-2021-N-176).

### Survey design

The survey was addressed to healthcare professionals working in Swiss clinics and hospitals treating stroke patients. Topics considered interesting by stroke patients and their carers have been previously investigated [20, 28], but for this survey healthcare professionals were chosen as target population. This approach was selected as they (i) are experts in the field, (ii) best know which type of information is usually provided to stroke patients, and (iii) know what information would be useful for stroke patients to reduce the risk of recurrent strokes and should, therefore, be prioritized in an educational program. Furthermore, stroke patients might not be familiar with rehabilitation technologies and could, consequently, struggle in answering questions on how these could be used to support their health education.

The survey consisted of a mix of multiple choice and open questions and was divided into four parts: First, demographics, where questions on specialisation and years of experience were asked. Second, the estimated level of stroke knowledge among stroke patients and current informing procedures. Here participants were asked to estimate stroke patients’ knowledge right after the first stroke and after spending some time in the healthcare facilities to evaluate the change in knowledge due to the information gathered during the rehabilitation journey. Additionally, four important stroke-related topics (i.e., risk factors for stroke, general health, treatment options, and stroke pathophysiology) were chosen based on literature and used to evaluate current information procedures. The topics regarding risk factors potentially leading to a second stroke and health in general were chosen as they may allow to minimize the risk factors of stroke [6, 9, 29] and possibly reduce the risk of recurrent strokes. Additionally, information about possible treatments and stroke pathophysiology were chosen as they may increase adherence to therapies [11] and have been identified as being of interest to stroke patients, respectively [28, 30]. Third, topics that stroke patients should be informed about and that should be included in an educational program. In this part, the lists of possible answers provided for the multiple-choice questions regarding relevant topics to include in an educational program and methods to deliver the information were selected based on literature [28, 30–34]. Furthermore, participants could specify additional points. Fourth, possible pathways for the implementation of an educational program using a rehabilitation technology were queried. The following indications were given in part 4 of the survey to help participants understand the general idea behind this project and how we foresee the implementation of health educational components as part of a rehabilitation technology: “Consider a rehabilitation device with a computer and a screen connected to it. This means, that you can use it to show text, videos, play audio files and that the patient can interact with it via a keyboard. Our idea is to display the information intermittently throughout the therapy, e.g., during breaks between exercises. We would provide one fact at a time, so that the patient can understand it in about 1 minute”. Indeed, being connected to a screen is the only requirement for a rehabilitation technology to provide educational information. To investigate whether it could be feasible to integrate an educational program in a technology-assisted rehabilitation session, participants were asked to indicate the number of minutes to dedicate to information in a 45-minute therapy session (0 corresponded to not wanting to integrate the educational program into the session). To address the problem of low awareness of personal risk for a second stroke, the stroke risk quiz developed by the American Heart Association [35] could be added to the educational program. A question about this point was therefore added to the survey. Other questions related to how to implement an educational program using rehabilitation technology were based on literature identifying important factors to promote satisfaction and retention of such educational information [20, 36] as well as practical aspects identified by the authors as critical for the implementation of such a program.

Since stroke patients can have a wide range of cognitive impairments, survey participants were asked to focus on stroke patients with no to mild cognitive impairment, as they would benefit most from an educational program. Educational program was defined as “the set of information we would like to give during the therapy sessions”.

### Survey validation

The survey was first developed in English. Face validity was ensured by having three researchers expert in the field of neurology and stroke rehabilitation look at the survey to evaluate whether the questions were considered relevant, and one expert on survey design check that the questions were properly formulated. The updated questionnaire was then translated into German, French, and Italian (i.e., three of the Swiss national languages) by native speakers and implemented on QuestionPro [37], an online software for surveys. To ensure that the survey was easy to understand for the target population and free of errors, a pilot evaluation was conducted. During the pilot evaluation, two healthcare professionals (one Italian-speaking medical doctor and one German-speaking physiotherapist) filled in the survey in their respective native languages and gave further input, e.g., on the clarity of the questions and correct use of medical terms. The final version of the survey consisted of 26 questions (Q1-Q26). The English version is provided in the Appendix (Additional File 1).

### Sample and distribution

The target population of the survey was healthcare professionals working in Swiss clinics and hospitals in which stroke patients are admitted and treated. We aimed to include different health-related professions as healthcare professionals with different roles interact in different ways with patients and might have different opinions about factors relevant for stroke education. The statistically significant minimum number of participants was calculated with an online sample size calculator [38] and defined as 150. Input parameters were 95% for the confidence level, 8% for the margin of error, and 120’214 for the population size. The population size is overestimated as it was determined by summing the number of doctors, nursing and social services staff, and personnel of other medical departments working in Swiss general hospitals and special clinics [2]. This corresponded to the total hospital personnel, not only to the one related to stroke, as it was not possible to identify the latter from the available statistics.

The link to the online survey was distributed via email to cantonal hospitals and private clinics in Switzerland treating stroke patients. Emails were sent to the general contact addresses of the facilities, with the request to forward the link to the relevant departments.

### Data analysis

The raw data of the survey were exported from QuestionPro and analysed with Matlab R2021b (MathWorks, Inc.). Participants who did not complete the demographics portion of the survey were excluded as it was considered that they dropped out of the survey before answering any relevant questions. Participants not primarily working in Switzerland were also excluded, as they did not correspond to our target population.

When relevant, answers were grouped according to participants’ role in the healthcare sector, i.e., medical doctor, physiotherapist, occupational therapist, speech therapist, nurse, neuropsychologist, social worker, or other. Descriptive statistics (mean (std)) and histograms were used to describe the data.

## Results

The survey was open from November 2021 to January 2022. In total, 196 responses were obtained. Thirty-five participants (17.9%) did not complete the demographics portion and were excluded. All of the remaining 161 respondents worked primarily in Switzerland and were considered for analysis; of these, 94 (58.4%) completed the entire survey. Due to this, the number of respondents per question varies as all responses collected were included in the analysis.

**Fig. 1:**
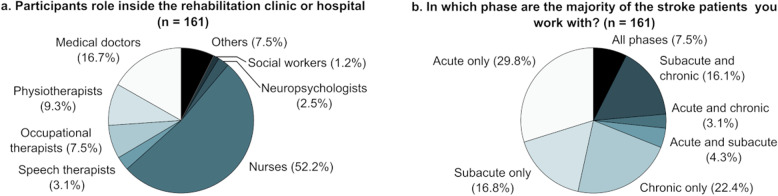
Participants’ role in the healthcare sector (**a**) and the stroke phase of the patients that the survey participants work with (**b**). n: number of respondents answering the question

Participant demographics are detailed in Fig. 1a. The mean number of years of general working experience was 13.9 (9.8). Participants worked with patients in different stages of stroke (Fig. 1b). Ninety-three participants (57.8%) were working with stroke patients daily, 34 (21.1%) on a weekly basis, 13 (8.1%) on a monthly basis, and 21 (13.0%) were not working with stroke patients on a regular basis. Participants working with stroke patients had on average 10.6 (8.2) years of experience working with them.

### Estimated level of stroke knowledge among stroke patients and current information procedures

**Fig. 2:**
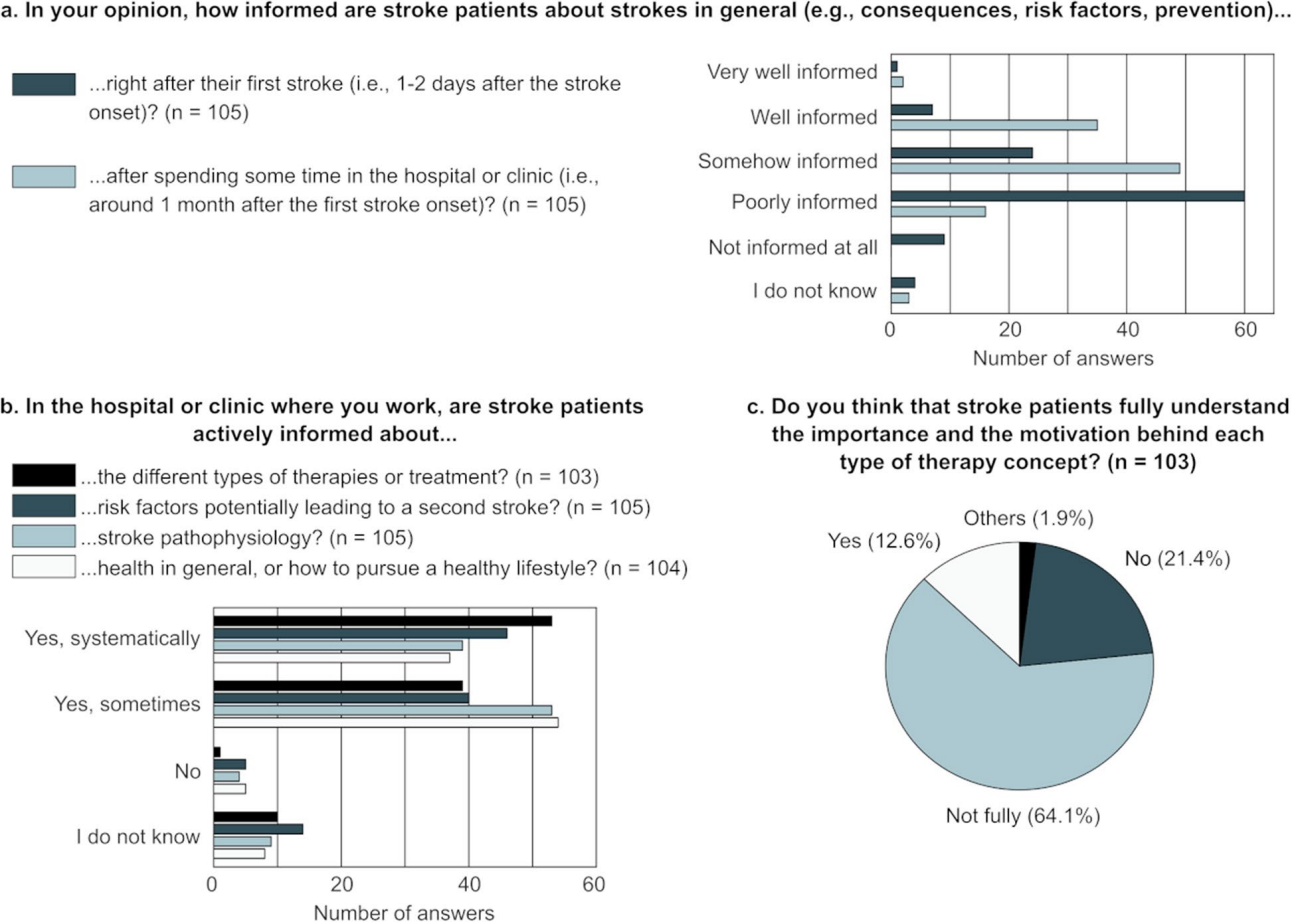
Estimated level of stroke knowledge among stroke patients at two different timepoints in the rehabilitation journey (**a**), frequency with which stroke patients are informed about four topics relevant to stroke during their stay in the health facilities (**b**), and estimated knowledge of the patients regarding the different therapy concepts and treatments (**c**). For (**c**), the two participants who answered “Other” provided “it varies from patient to patient, depending on the initial care provided in the acute and subacute phase” and “yes, but not in the immediate post-stroke or acute phase” as alternative answers. n: number of respondents answering the question

The level of stroke knowledge among stroke survivors estimated by the healthcare professionals participating in this survey is shown in Fig. 2a.

The frequency with which stroke patients are informed about four relevant, stroke-related topics in the clinical routine is depicted in Fig. 2b (Q21-24). If the answer to Q21-Q24 was “Yes”, participants had to specify the methods employed to inform patients about the different topics (Table 1). Most of the additional methods listed when answering “Other” could be categorized as “Visits from a healthcare professional to discuss topics specific to the stroke patient” and “Dedicated sessions held by a healthcare professional to inform one or a group of stroke patients about a predefined topic”. A method not listed but mentioned by the participants (*n* = 3) was delivering information spontaneously during the various interactions between healthcare professionals and patient, e.g., in response to questions asked by the patient.

Among those who answered that stroke patients are not actively informed about stroke pathophysiology, some stated that it was because “patients in the chronic stage do not raise these questions” (*n* = 1) and that “there are no specific moments dedicated to this, but patients receive appropriate answers from the various professionals according to their situation and questions” (*n* = 1). Three nurses working with acute stroke patients reported that they do not inform patients about risk factors and health in general because acute stroke patients have poor learning ability and that in this phase the priority is treatment. Other explanations mentioned were that this type of information “is not well accepted by patients” (*n* = 1), that often nurses and assistant physicians are not well informed about the link between risk factors and stroke prevention (*n* = 1), “because nobody feels responsible” (*n* = 1), or that “there is no specific procedure” (*n* = 1). The estimated knowledge about different therapy concepts and treatments (Q25) is shown in Fig. 2c.

**Table 1:** Number of responses (percentage respondents) regarding the methods used to deliver information in clinical practice

	Therapy and treatments* (*n* = 92)		Stroke risk factors* (*n* = 84)		Stroke pathophysiology* (*n* = 91)		Health in general* (*n* = 90)	
Visits from a healthcare professional to discuss topics specific to the stroke patient	78	(84.8%)	64	(76.2%)	67	(73.6%)	67	(74.4%)
Brochures that patients read on their own	26	(28.3%)	36	(42.9%)	39	(42.9%)	36	(40.0%)
Dedicated sessions held by a healthcare professional to inform one or a group of stroke patients about a predefined topic	27	(29.3%)	25	(29.8%)	25	(27.5%)	29	(32.2%)
Brochures that patients read with the help of a healthcare professional	16	(17.4%)	18	(21.4%)	12	(13.2%)	12	(13.3%)
Videos that patients watch on their own	3	(3.3%)	6	(7.1%)	7	(7.7%)	3	(3.3%)
Books that patients read on their own	4	(4.3%)	4	(4.8%)	5	(5.5%)	6	(6.7%)
Books that patients read with the help of a healthcare professional	0	(0.0%)	1	(1.2%)	1	(1.1%)	0	(0.0%)
Videos that patients watch with a healthcare professional	1	(1.1%)	1	(1.2%)	2	(2.2%)	0	(0.0%)
Other	3	(3.3%)	10	(11.9%)	11	(12.1%)	4	(4.4%)

*n *Number of respondents answering the question

*Each respondent could choose multiple options

### Key topics for an educational program for individuals with stroke

**Fig. 3:**
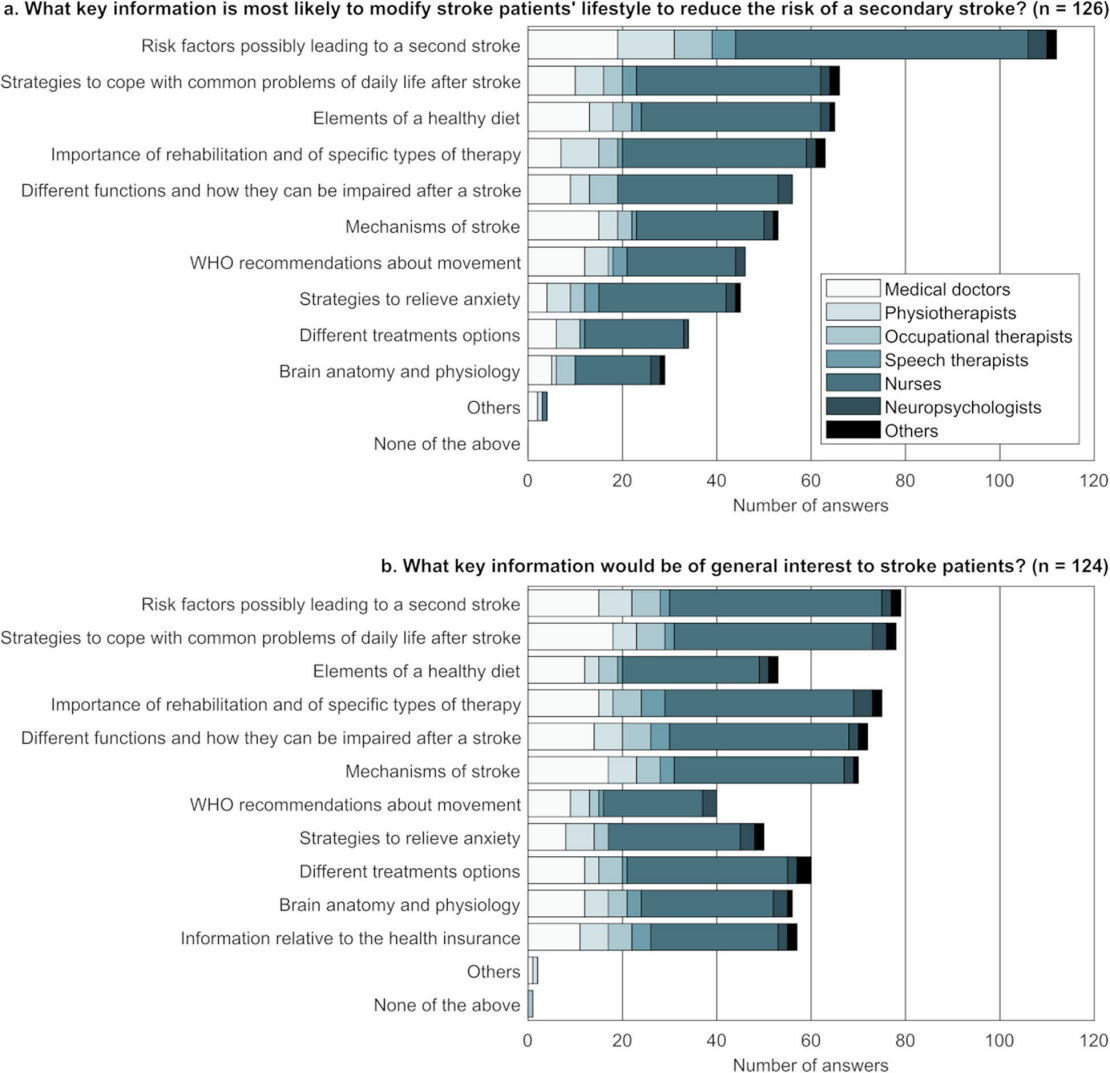
Topics that are most likely to reduce the risk of a secondary stroke (**a**) or that are considered interesting for patients (**b**) according to each of the roles covered by the respondents of this survey (no social workers provided an answer to these two questions). Participants could select multiple answers. n: number of respondents answering the question

The topics that are most likely to reduce the risk of a secondary stroke (Q8) or that are considered interesting for patients (Q9) according to the survey respondents are shown in Fig. 3. Other topics not listed as possible answers to the question but suggested by the participants as most likely to reduce the risk of a secondary stroke were “help to stop smoking” (*n* = 1) and “physical activities that can be performed without a therapist” (*n* = 1). Additional topics suggested as being of general interest for stroke patients included “different state, parastatal and private structures that exist in the area and that can be indispensable or of great help” (*n* = 1) and “information on equipment related to physical activity that can be reimbursed by the health insurance” (*n* = 1).

Seventy-three (56.6%) participants strongly agreed with the fact that being better informed about the reason behind a given type of therapy would further motivate stroke survivors during rehabilitation (Q10), 49 (38.0%) agreed, 1 (0.8%) neither agreed nor disagreed, 5 (3.9%) disagreed, and 1 (0.8%) strongly disagreed.

### Implementing an educational program on a rehabilitation technology

Overall, the preferred mean time to dedicate to education in a 45-minute robot-assisted therapy session was 10.8 (7.0) minutes (Q18, *n* = 106). Specifically, the preferred mean time was of 8.3 minutes for speech therapists and neuropsychologists, 8.7 minutes for occupational therapists, 10.1 minutes for medical doctors, 10.5 minutes for physiotherapists, and 11.4 minutes for nurses.

**Fig. 4:**
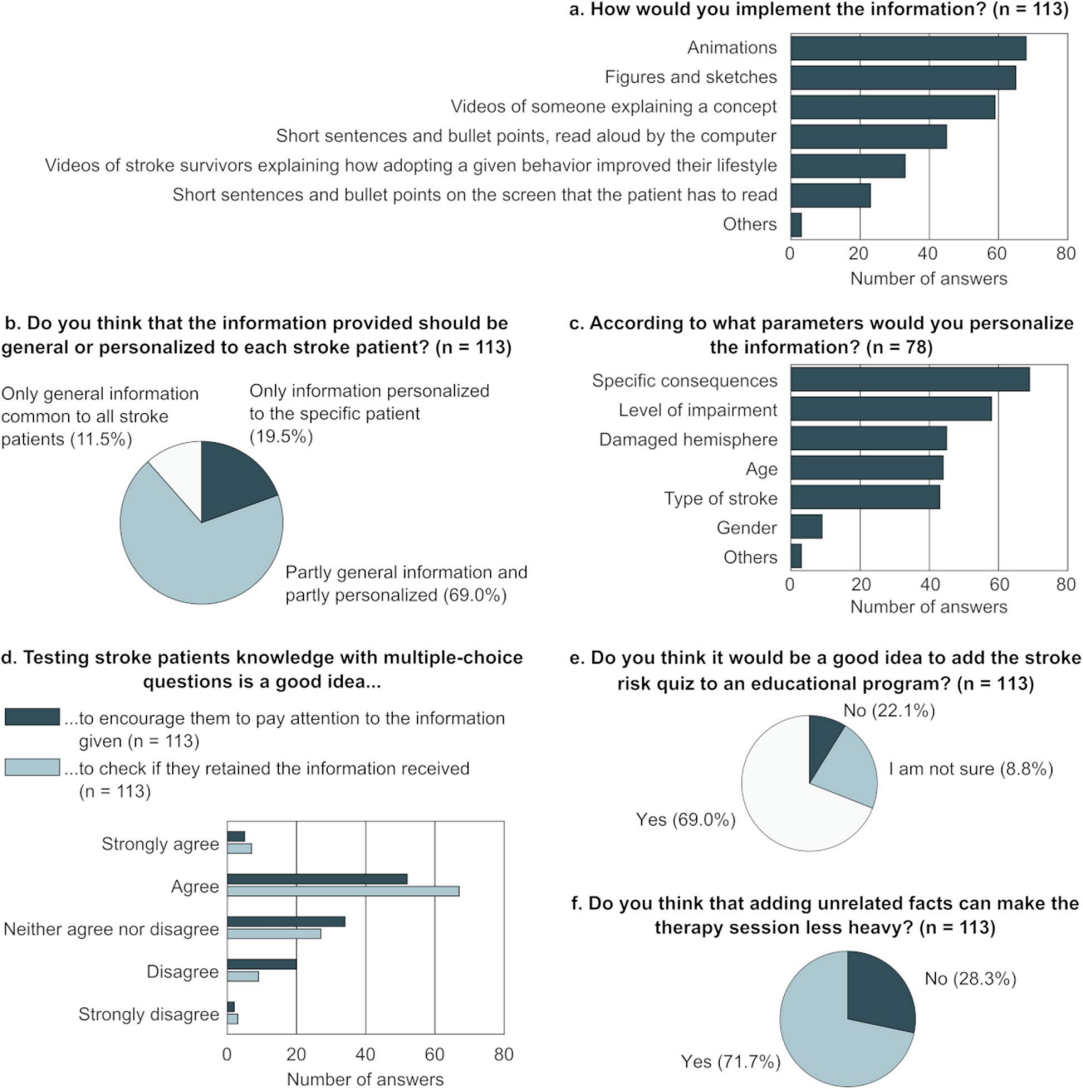
Preferred methods for delivering the information to stroke patients according to healthcare professionals (**a**). Participants could tick a maximum of three options. Participants’ opinion about personalizing the information (**b**) and the factors to consider when doing this (**c**) are also shown, together with results about adding multiple choice questions (**d**), unrelated facts (**e**), and the stroke risk quiz (**f**). n: number of respondents answering the question

The preferred methods for delivering the information (Q11) are shown in Fig. 4a. The three participants answering “Other” mentioned “alternating videos, animations or sentences could keep the attention high and adapt more to one concept or another”, “explanations by a trained and specialized stroke education health professional (nurse or physician)”, and “information to be customised according to the patient’s understanding” as additional points to consider.

Most participants stated that the delivered information should be personalized (Fig. 4b) and “specific consequences (e.g., hemineglect, diplopia)” was the most frequently chosen factor when considering how to personalize the information (Fig. 4c, Q14-Q15). Additional factors suggested by the participants were “profession pursued or to be pursued, hobbies, and other interests” (*n* = 1), “type of speech disorder (if any)” (*n* = 1), and “education” (*n* = 1).

Forty-six percent of the participants agreed on multiple-choice questions being a good idea to encourage patients to pay attention to the information given, while 59.3% agreed on multiple-choice questions being a good approach to check if patients retained the information received (Fig. 4d, Q12-Q13).

Adding a stroke risk quiz to assess patients’ personal risk of stroke to increase awareness was considered a good idea by 78 (69.0%) participants (Fig. 4e, Q16). The reasons listed by participants against or unsure about the quiz were that such a quiz might be too difficult for patients to understand and fill in (*n* = 14), can induce negative states of mind (e.g., anxiety, guilt, confusion; *n* = 11), and that it is not useful to induce a change in awareness or behaviour (*n* = 3).

Eighty-one (71.7%) participants confirmed that adding humorous or interesting unrelated facts can make the therapy session embedding the educational program less heavy (Fig. 4f, Q17). The reasons mentioned by those who opposed the idea were that unrelated facts are a distraction from the important topics or could overload patients (*n* = 21), are simply not relevant or not needed (*n* = 5), would make the therapy session too long (*n* = 3), can create confusion or reduce the credibility of the intervention (*n* = 3), and that humour is not for everyone (*n* = 1).

## Discussion

Health information after stroke is important as it might decrease the risk of recurrent strokes, and improve functional outcomes [10]. Technology-based education has been proposed as a possible way to support the delivery of health information and studies reported that stroke patients are interested in it [20]. However, how rehabilitation technologies could best help inform patients is still to be extensively explored, especially in terms of information content and methods of information delivery. The main goal of this work was, therefore, to evaluate health knowledge after stroke by gathering the opinion of healthcare professionals working in Swiss hospitals and rehabilitation clinics admitting stroke patients. Specifically, this survey investigated the estimated level of knowledge of stroke patients regarding their disease, how stroke patients are informed during the rehabilitation journey, the topics that should be covered in an educational program, and finally whether such an educational program could be embedded into a rehabilitation technology and how it should be implemented.

The participants of the survey represented the population that we originally targeted, as the respondents were working in different parts of Switzerland (as deduced from the answers given in 3 different languages) and 87% were working on a regular basis with stroke patients in all phases of stroke. Moreover, the distribution of the roles covered by the participants represented the general distribution of roles in the Swiss healthcare facilities, which shows a higher number of nurses compared to medical doctors or other medical departments [2]. Furthermore, nurses are likely the ones best informed about the general health situation and knowledge of patients.

According to the survey respondents, stroke patients’ level of knowledge could be improved, and technologies can play a role in facilitating this process.

### There is room for improvement in stroke and health education among stroke patients according to healthcare professionals

The results of this survey confirmed what is generally found in the literature, namely that there is room for improvement with regards to the information level of stroke patients about their health and disease. The majority of the survey participants stated that, right after stroke, patients are poorly informed about their disease. A poor knowledge of risk factors early after hospitalization was also identified by other groups [13, 39] and may be linked to the suboptimal health literacy among elderly or the general population [40]. This supports the idea that it would be useful to make educational programs accessible to all individuals at risk or to the population in general (i.e., primary prevention), not only to people who have already suffered a stroke [41], and technology-based unsupervised education (e.g., via a smartphone app) would be a good solution for this application, as it would allow people who are not hospitalised or not regularly followed by healthcare professionals to have access to relevant information in a controlled manner.

When comparing the estimated level of knowledge of patients right after stroke and after spending some time in the rehabilitation facilities, there is a shift towards being better informed. However, only 33% of healthcare professionals thought that patients are well informed, even at the latest timepoint, which is again in line with previous literature [42]. Ninety-four percent of the survey participants agreed that being better informed about the reasons behind a given type of therapy would further motivate stroke survivors during rehabilitation. However, 64% of participants stated that stroke patients do not fully understand the motivation behind the different types of therapy they engage in, which is supported by previous work showing that stroke patients have poor treatment knowledge [13].

### An educational program should cover different topics and be personalized

As could be expected, 88.9% of the participants selected “risk factors for stroke” as the key topic most likely to modify patients’ lifestyle and reduce the risk of a second stroke. The distributions of the topics identified as important to reduce the risk of a second stroke and of those selected as being of general interest to stroke patients did not align. Specifically, topics selected as being of general interest were more homogeneous across respondents. This is in line with the findings from another study [34] describing that what stroke patients reportedly would like to be informed about did not always match the topics selected as relevant for them by medical professionals. For example, in contrast to doctors and nurses, patients prioritized information about diet management over risk factors, which was suggested to be a consequence of a lack of awareness about their importance.

The majority of the respondents stated that the information delivered with the help of a rehabilitation technology should be personalized according to different factors, of which specific consequences of stroke (e.g., hemineglect, diplopia) and the level of impairment were the preferred ones. It would therefore seem necessary to develop a modular educational program, as suggested in [20] and implemented as a personalized booklet containing general and patient-specific information in [43]. A personalized selection of relevant topics might additionally promote motivation to follow the program.

### Technologies and multimedia material that support health information are not commonly used

According to the survey responses, the most common information method used in clinical practice was visits from a healthcare professional to discuss topics specific to the stroke patient, followed by brochures and dedicated sessions for one or a group of patients, which aligns with the literature [44]. In contrast, the use of videos was indicated as one of the least used methods. However, videos and animations were in the top three methods chosen by the survey participants as preferred to deliver technology-based education. This discrepancy highlights the need to develop multimedia material to support information and to revisit how information is delivered with the use of new tools. Furthermore, the wealth of solutions offered by digital technologies seems not yet to be widely used. A possible reason might be that they might not be trusted, as not so well validated. However, nowadays most patients in Switzerland own smartphones or laptops that could support apps for health education. In addition, rehabilitation technologies are increasingly widespread. Therefore, the possibility of using technologies to increase health literacy exists but is not yet exploited by clinicians or those who develop technology for rehabilitation.

### Integrating health information in technology-assisted rehabilitation sessions is perceived as an acceptable option

Dedicating about 10 min of a 45-minutes technology-assisted rehabilitation session to education was considered generally acceptable by participants covering different roles inside the healthcare facilities. This number is slightly higher than the time dedicated to stroke education in [26] and [25], where 5 min of information were integrated in technology supported supervised and unsupervised rehabilitation sessions.

However, more than just providing the information, patients need to retain the information. Sixty-five percent of the respondents agreed that multiple-choice questions could be integrated into an educational program to check if the information is retained. The answers to those questions could then be used to define if, for a given patient, any information needs to be repeated. The idea of increasing patients’ awareness about their risk factors by integrating a quiz developed to assess the personal risk of stroke [35] was positively received by the majority of the respondents. However, around 20% of the survey respondents raised concerns regarding the understandability of the quiz and the triggering of negative emotions such as fear or guilt. These factors should be considered with the possibility that the quiz would only be added for certain patients based on a clinician’s decision.

More than 70% of the respondents also agreed on adding humorous or interesting unrelated facts to the therapy session to make it more enjoyable. The arguments against this idea brought by the other participants were mainly related to these facts being a distraction from the relevant topics and that patients often have cognitive deficits, so processing this additional information could cause an unnecessary overload. Unrelated facts could, therefore, be integrated into the educational program only for patients with minor cognitive deficits and it should be possible to remove them if the patient requests it.

### Limitations and outlook

While this work allowed to identify ways to improve health education after stroke, three main limitations must be acknowledged. First, despite the number of individuals starting the survey being higher than our target sample size, the number of participants who completed the whole survey was lower. However, the target sample size was calculated based on the total number of healthcare professionals working in Switzerland, not only those working with stroke patients. Therefore, the initial target sample size was overestimated, and consequently the sample size of this survey should still be representative.

Second, despite the survey targeting the health knowledge of stroke patients, we chose not to involve them in the survey, and we did not directly test their knowledge. Instead, this work was based on the perception that healthcare professionals have about stroke patients’ knowledge. However, the respondents of this survey had on average 10 years of experience in working with stroke patients and could, therefore, be considered experts in the field with a clear idea of the topics covered here. Furthermore, healthcare professionals are the ones that can judge what a patient should know or should be informed about, as patients might not know what the important topics are or what rehabilitation technologies are or could provide. As such, it would have been difficult for patients to answer such a survey in a meaningful way.

Finally, the survey was limited to Switzerland, therefore, results concerning the level of patients’ knowledge and current practice in delivering information might differ from other countries. Nonetheless, we expect the results regarding methods and factors to be considered in the implementation of an educational program in a rehabilitation technology to be generalisable.

## Conclusions

This work suggests that the level of stroke knowledge of stroke patients can be improved and that technology can play a role in this. Furthermore, this work helped to identify topics of key importance for the implementation of an educational program and the methods that could be used when implementing it in the context of technology-supported rehabilitation.

## Supplementary Information

Supplementary Material 1. *Devittori2024_BMCPublicHealth_Additional File 1_Survey.pdf*. Questions included in the survey administered online to study participants.

## Data Availability

The datasets generated during the study detailed here will be available from the corresponding author upon reasonable request.

## Abbreviations

ETH: Swiss Federal Institute of Technology
Qn: Question number n
