# Supplementary material for: Health knowledge after stroke in Switzerland: a survey among health professionals on current practice and suggestions for the implementation of a technology-based educational program for stroke survivors
Source: BMC Health Serv Res. 2024 Oct 19;24:1259. doi: 10.1186/s12913-024-11735-0 (PMC11490157; doi:10.1186/s12913-024-11735-0)
Supplement: Supplementary file 1 — Supplementary Material 1. Devittori2024_BMCPublicHealth_Additional File 1_Survey.pdf. Questions included in the survey administered online to study participants. [file 12913_2024_11735_MOESM1_ESM.pdf]

Health knowledge after stroke in Switzerland: A survey on current practice and suggestions for the implementation of a technology-based educational program

Devittori et al., BMC Public Health, 2024

Appendix 1

A. Demographics

1. What is your main role inside the rehabilitation clinic or hospital?
    1. Medical doctor*
    2. Physiotherapist
    3. Occupational therapist
    4. Neuropsychologist
    5. Nurse
    6. Nutritionist
    7. Speech therapist
    8. Social worker
    9. Other (please specify) __________

    *Specialty: _________________________

2. How many years have you been doing this job (approximately)?
    - *Possible answers ranged from 1 to 70*

3. What country do you primarily work in?
    1. Switzerland
    2. Italy
    3. Germany
    4. France
    5. Austria
    6. Other (please specify) __________

4. Do you work with stroke patients on a regular basis?
    1. Yes, at least once per day
    2. Yes, at least once per week
    3. Yes, at least once per month
    4. No

5. How many years have you been working with stroke patients (approximately)?
    - *Possible answers ranged from 1 to 70*

6. In which phase are the majority of the stroke patients you work with?
    1. Acute (between 24 hours and 1 week after stroke onset)
    2. Subacute (between 1 week and 3 weeks after stroke onset)
    3. Chronic (more than 3 weeks after stroke onset)

7. Are you involved in any research activities at your institution (e.g., development or validation of new technologies, clinical trials)?
   1. Yes
   2. No

B. Key information for an educational program

Consider that the target population for the educational program we want to implement consists of stroke patients with no to mild cognitive impairments. Therefore, when answering the questions below, please do not refer to patients with moderate or severe cognitive impairments. Note: by "educational program" we mean the set of information we would like to give during the therapy sessions.

8. What key information is most likely to **modify stroke patients' lifestyle to reduce the risk of a secondary stroke**? Assume that this information will be explained in a simplified and understandable way.
   1. Brain anatomy and physiology (e.g., brain regions and their functions, why strokes can lead to various outcomes)
   2. Mechanisms of stroke (e.g., ischemic vs. haemorrhagic, pathological mechanisms)
   3. Different functions and how they can be impaired after a stroke (e.g., motor functions, sensory functions, importance of sensory functions in fine movements)
   4. Importance of rehabilitation and of specific types of therapy
   5. Different treatment options
   6. Risk factors possibly leading to a second stroke
   7. Elements of a healthy diet
   8. World Health Organization recommendations for the amount of movement per day
   9. Strategies to relieve anxiety
   10. Strategies to cope with common problems of daily life after stroke
   11. None of the above
   12. Others (please add any relevant information) __________

9. What key information would be of **general interest to stroke patients**? Let's assume that this information will be explained in a simplified and understandable way.
   1. Brain anatomy and physiology (e.g., brain regions and their functions, why strokes can lead to various outcomes)
   2. Mechanisms of stroke (e.g., ischemic vs. haemorrhagic, pathological mechanisms)
   3. Different functions and how they can be impaired after a stroke (e.g., motor functions, different sensory functions, importance of sensory functions in fine movements)
   4. Importance of rehabilitation and of a specific type of therapy
   5. Different treatment options
   6. Risk factors possibly leading to a second stroke
   7. Elements of a healthy diet
   8. World Health Organization recommendations for the amount of movement per day
   9. Strategies to relieve anxiety
   10. Strategies to cope with common problems of daily life after stroke
   11. Information relative to the health insurance (e.g., which assistive devices are supported (wheelchair, walker, …), number of supported therapies after discharge)
   12. None of the above
   13. Others (please add any relevant information) __________

10. State your level of agreement with the following statement: “Being better informed about the reason behind a given type of therapy would further motivate stroke survivors during rehabilitation”
    1. Strongly disagree
    2. Disagree
    3. Neither agree nor disagree
    4. Agree
    5. Strongly agree

C: Implementing an educational program on a rehabilitation device

Consider a rehabilitation device with a computer and a screen connected to it. This means, that you can use it to show text, videos, play audio file and that the patient can interact with it with a keyboard. Our idea is to display the information intermittently throughout the therapy, e.g., during breaks between exercises. We would provide one fact at a time, so that the patient can understand it in about 1 minute.

11. How would you implement the information discussed before (tick a maximum of 3 options)?
    1. Short sentences and bullet points on the screen that the patient has to read
    2. Short sentences and bullet points on the screen, read aloud by the computer
    3. Figures and sketches that illustrate the concept
    4. Animations
    5. Videos of someone explaining a concept
    6. Videos of stroke survivors explaining how adopting a given behaviour improved their lifestyle (e.g., how quitting smoking improved their health)
    7. Other(please specify) __________

Furthermore, we are considering implementing multiple-choice questions (4 possible answers) to test the retention of information and to encourage stroke patients to pay attention to the information provided.

12. State your level of agreement with the following statement: “Testing stroke patients’ knowledge with multiple-choice questions is a good idea to **encourage them to pay attention** to the information given”
    1. Strongly disagree
    2. Disagree
    3. Neither agree nor disagree
    4. Agree
    5. Strongly agree

13. State your level of agreement with the following statement: “Testing stroke patients’ knowledge with multiple-choice questions is a good idea to **check if they retained** the information received”
    1. Strongly disagree
    2. Disagree
    3. Neither agree nor disagree
    4. Agree
    5. Strongly agree

14. Do you think that the information provided should be general or personalized to each stroke patient?
    1. Only general information common to all stroke patients
    2. Partly general information and partly personalized
    3. Only information personalized to the specific patient

15. According to what parameters would you personalize information?
    1. Age
    2. Type of stroke (e.g. ischemic vs haemorrhagic)
    3. Damaged hemisphere
    4. Gender
    5. Level of impairment
    6. Specific consequences (e.g., hemineglect, diplopia)
    7. Other(please specify) __________

16. The American Stroke Association developed a “Stroke Risk Quiz” that assesses the risk of stroke based on questions about risk factors (see figure). Do you think it would be a good idea to add this to an educational program to make people even more aware of their personal risk level?
    1. Yes
    2. I’m not sure*
    3. No*

    *Why? Please specify: ____

To make therapy a bit more entertaining, and not to focus only on information related to stroke and health, we are considering adding humorous, cute or interesting unrelated facts to the information provided (which will not be tested). For example: did you know that otters sleep in the sea, and hold hands while sleeping to prevent themselves from floating away?

17. Do you think that adding these unrelated facts can make the therapy session less heavy?
    1. Yes
    2. No*

    *Why not? (Please explain): ____

The idea is to provide about 1-2 pieces of information and 1-2 questions per therapy session, which usually lasts about 45 minutes.

18. In your opinion, in a therapy session of 45 minutes focusing on robot-assisted hand rehabilitation, how much time should be spent on the educational program?
    - *Possible answers ranged from 0 to 45*

D. Current information procedures

19. In your opinion, how informed are stroke patients about strokes in general (e.g., consequences, risk factors, prevention) **right after their first stroke** (i.e., 1-2 days after the stroke onset)?
    1. Very well informed (patients memorized all the relevant information)
    2. Well informed
    3. Somehow informed
    4. Poorly informed
    5. Not informed at all
    6. I don't know

20. In your opinion, how informed are stroke patients about strokes in general (e.g., consequences, risk factors, prevention) **after spending some time in the hospital or clinic** (i.e., around 1 month after the first stroke onset)?
    1. Very well informed (patients memorized all the relevant information)
    2. Well informed
    3. Somehow informed
    4. Poorly informed
    5. Not informed at all
    6. I don't know

21. In the hospital or clinic where you work, are stroke patients actively informed about **stroke pathophysiology** (e.g., "ischemic stroke is caused by deficient blood and oxygen supply to the brain")?
    1. Yes, systematically (as a standard procedure applied to every patient)
    2. Yes, sometimes (only if a patient asks, or only to some patients)
    3. No
    4. I don't know

    If yes, how?
    1. Visits from a healthcare professional to discuss topics specific to the stroke patient
    2. Dedicated sessions held by a healthcare professional to inform one or a group of stroke patients about a predefined topic
    3. Brochures that patients read on their own
    4. Brochures that patients read with the help of a healthcare professional
    5. Books that patients read on their own
    6. Books that patients read with the help of a healthcare professional
    7. Videos that patients watch on their own
    8. Videos that patients watch with a healthcare professional
    9. Other (please add any further procedure used) __________

    If not, why not? Please explain: ____________________________

22. In the hospital or clinic where you work, are stroke patients actively informed about **risk factors potentially leading to a second stroke**?

1. Yes, systematically (as a standard procedure applied to every patient)
2. Yes, sometimes (only if a patient asks, or only to some patients)
3. No
4. I don't know

If yes, how?

1. Visits from a healthcare professional to discuss topics specific to the stroke patient
2. Dedicated sessions held by a healthcare professional to inform one or a group of stroke patients about a predefined topic
3. Brochures that patients read on their own
4. Brochures that patients read with the help of a healthcare professional
5. Books that patients read on their own
6. Books that patients read with the help of a healthcare professional
7. Videos that patients watch on their own
8. Videos that patients watch with a healthcare professional
9. Others (please add any further procedure used) __________

If not, why not? Please explain: ____________________________

23. In the hospital or clinic where you work, are stroke patients actively informed about **health in general**, or how to pursue a **healthy lifestyle**?

1. Yes, systematically (as a standard procedure applied to every patient)
2. Yes, sometimes (only if a patient asks, or only to some patients)
3. No
4. I don't know

If yes, how?

1. Visits from a healthcare professional to discuss topics specific to the stroke patient
2. Dedicated sessions held by a healthcare professional to inform one or a group of stroke patients about a predefined topic
3. Brochures that patients read on their own
4. Brochures that patients read with the help of a healthcare professional
5. Books that patients read on their own
6. Books that patients read with the help of a healthcare professional
7. Videos that patients watch on their own
8. Videos that patients watch with a healthcare professional
9. Others (please add any further procedure used) __________

If not, why not? Please explain: ____________________________

24. In the hospital or clinic where you work, are stroke patients actively informed about the **different types of therapies or treatments**?
    1. Yes, systematically (as a standard procedure applied to every patient)
    2. Yes, sometimes (only if a patient asks, or only to some patients)
    3. No
    4. I don't know

    If yes, how?
    1. Visits from a healthcare professional to discuss topics specific to the stroke patient
    2. Dedicated sessions held by a healthcare professional to inform one or a group of stroke patients about a predefined topic
    3. Brochures that patients read on their own
    4. Brochures that patients read with the help of a healthcare professional
    5. Books that patients read on their own
    6. Books that patients read with the help of a healthcare professional
    7. Videos that patients watch on their own
    8. Videos that patients watch with a healthcare professional
    9. Others (please add any further procedure used) __________

    If not, why not? Please explain: ____________________________

25. Do you think that stroke patients fully understand the importance and the motivation behind each type of therapy concept (e.g., physiotherapy method used, medical training therapy (MTT), diet, …)?
    1. Yes, I think that in general stroke patients are aware of the importance and motivation of the different therapies. They are able to explain these concepts in a complete and correct way to someone else
    2. I think that in general stroke patients are aware of the importance of the types of therapy, but they do not fully understand the motivation behind it. They would not be able to explain these concepts to someone else
    3. No, I think that in general stroke patients do therapy because they are told to, without understanding the motivation
    4. Other (please specify) __________

26. Do you have any other general comments, thoughts, or suggestions?
    1. No
    2. Yes __________
